# Effect of Amplatz Sheath on Cystolithotripsy for Women with Large Bladder Stone

**DOI:** 10.1155/2017/9341042

**Published:** 2017-05-24

**Authors:** Tadashi Tabei, Takashi Kawahara, Shinnosuke Kuroda, Hiroki Ito, Kazuki Kobayashi, Hiroji Uemura, Junichi Matsuzaki

**Affiliations:** ^1^Department of Urology, Yokosuka Kyosai Hospital, Yokosuka, Japan; ^2^Department of Urology and Renal Transportation, Yokohama City University Medical Center, Yokohama, Japan; ^3^Department of Urology, Yokohama City University Graduate School of Medicine, Yokohama, Japan; ^4^Department of Urology, Ohguchi Higashi General Hospital, Yokohama, Japan

## Abstract

**Objective:**

This study compared the effect of endourological procedures with or without the Amplatz sheath (AS) on cystolithotripsy.

**Methods:**

We retrospectively analysed 18 patients who underwent treatment for bladder stone over 30 mm. This study consisted of two groups, namely, patients who underwent cystolithotripsy with an AS (AS group) and those who underwent standard procedure without an AS (SP group). The stone-free rate, total energy used for operation, operation time, days of admission after operation, and complication of both groups were compared.

**Results:**

The number of patients in the AS and SP groups was 10 and 8, respectively. Significant differences were not found between these two groups with regard to age, stone burden, stone volume, number of stones, and history of neurogenic bladder. All patients in both groups achieved a stone-free state. Total energy was significantly increased and operation time was shorter in the AS group. No significant difference was observed in terms of days of admission after operation. Any complications were not increased by the use of AS. Struvite was the most common stone component in both groups.

**Conclusion:**

Use of an AS can shorten the operation time of cystolithotripsy without increasing perioperative complication.

## 1. Introduction

A number of treatment options are available for bladder stones including shock wave lithotripsy (SWL), transurethral cystolithotripsy, percutaneous lithotripsy, and open surgery. Endoscopic transurethral cystolithotripsy is among the most well-known procedures for the majority of urologists. However, disintegrating and extracting stones from the bladder, particularly in cases of large stones, take a considerable amount of time with usual cystolithotripsy.

Maheshwari reported a novel technique in 1998 [1]. He used periurethral Amplatz sheath (AS), which greatly reduced the time needed for the entire cystolithotripsy procedure for 2 women with large bladder stone. Okeke et al. reported 5 cases of men treated with the AS [2]. Based on these reports, we also utilised the AS for cystolithotripsy of female patients with large bladder calculi since November 2011. Kawahara et al. also demonstrated 3 cases with similar conditions in 2012 [3]. To the best of our knowledge, no study has compared cystolithotripsy using AS with standard endourological procedure. Therefore, in the present study, we compared the effectiveness of two endourological treatments for large bladder stone on cystolithotripsy with or without an AS.

## 2. Materials and Methods

We retrospectively analysed 18 female patients who underwent treatment for bladder stone over 30 mm diameter between May 2009 and December 2014 at the Ohguchi Higashi General Hospital, Yokohama, Japan. We routinely utilised the AS for cystolithotripsy of large bladder stone since November 2011. This study consisted of patients who underwent standard procedure with an AS (AS group) and those without an AS (SP group). The stone-free rate, total energy used for operation, operation time, days of admission after operation, and complication of both groups were compared. Postoperative pain was compared by usage of analgesic until hospital discharge.

Stone burden was defined as the sum of the stones' maximum diameter on radiography of the kidneys, ureters, and bladder (KUB) films. Stone volume was calculated using the ellipsoid formula: height (mm) × width (mm) × depth (mm) ×  *π*/6 × 1/1000. Each diameter was measured in preoperative computed tomography. Stone-free status was assessed with a plain KUB film at postoperative day 1. This status was defined as no visible fragments on the film.

Written informed consent was obtained from all individual participants included in the study.

This study was conducted according to the Helsinki Declaration. The ethics committee of Kanagawa Prefecture Medical Association approved this study.

### 2.1. Surgical Technique of Cystolithotripsy with or without the AS

**Figure 1:**
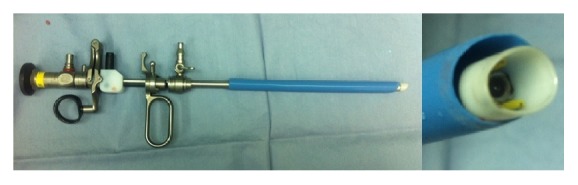
The 30 Fr Amplatz sheath and 24 Fr cystoscope. The 6 Fr difference between the two results in high supply of irrigation flow with low intravesicle pressure.

We previously reported the surgical procedure with an AS [3]. All patients were placed in the lithotomy position. We initially inserted a 24 Fr scope covered with a 26.5 Fr outer sheath and placed the AS smoothly over the outer sheath. We inserted a 550 *μ*m laser fibre covered with a cut 5 Fr ureteral catheter into the work channel. Stone fragments were disintegrated using 100 W laser generators (VersaPulse PowerSuite 100 W; LUMENIS Surgical, CA, USA) and then were removed by an irrigation flow through the AS. The irrigation flow was fixed at 100 mmHg after detaching the 26.5 Fr outer sheath (Figure 1). The laser power and rate were limited within 3.5 J and 30 Hz, respectively. Each operator could determine the laser setting within this limit as strong power or as frequent rate as visualisation was kept clear.

The surgical procedure without an AS, a group of standard procedure, is almost the same. However, a 26.5 Fr outer sheath was not detached in those cases because inserting a scope in and out through the urethra becomes more difficult without the outer sheath. Irrigation flow was maintained by natural pressure. The limitation of laser setting was the same as the AS group.

### 2.2. Statistical Analysis

Mann–Whitney test and *χ*^2^ test were performed to compare between these two groups. Continuous variables were expressed as median (minimum–maximum). Statistical significance was set at *P* < 0.05. Statistical analyses were carried out using the SPSS version 19 software package (SPSS, Chicago, IL, USA).

## 3. Results

The characteristics of patients are shown in Table 1. The number of patients was 10 in the AS group and 8 in the SP group. No significant differences were found between the two groups with regard to age, stone burden, stone volume, number of stones, and history of neurogenic bladder and history of febrile urinary tract infection. All of 18 patients had preoperative pyuria. A stone-free status was achieved in all cases.  Table 2 shows the outcomes of each procedure. Total energy and operation time were significantly different between the two groups (total energy: 62.93 kJ versus 27.64 kJ *P* = 0.015; operation time: 58.50 minutes versus 112.0 minutes *P* = 0.015). No significant difference was found in terms of days of admission after operation. Complications such as mucosal injury, incontinence, and pain after operations were not observed. One patient in SP group experienced postoperative fever that was treated conservatively. Struvite was the most common stone component in both groups. No recurrence has been found although 7 of 18 patients were lost to follow-up.

## 4. Discussion

Bladder calculi represent 5% of all urinary stones in developed countries [4]. The majority of adult patients with bladder stone have several predisposing factors that promote stone formation, such as voiding dysfunction, bladder outlet obstruction, infection, and a foreign body (sutures, catheters, and self-introduced objects) [5]. Therefore, treatment should be performed with care of less residual fragments on the basis that spontaneous fragment passage is unlikely [6].

Several treatments are available for bladder stones including open surgery, SWL, percutaneous lithotripsy, and transurethral cystolithotripsy. Open surgery remains a standard treatment for paediatric bladder stone [7]. However, by improving endourological modality, this treatment is rare for adults. The indication of open surgery for adult bladder stone is not definite. Treatment of bladder stone larger than 4 cm is generally performed via open surgery [6].

SWL is sometimes performed because of its tolerance for high-risk patients [8]. However, it is not effective for large bladder stone because even if fragmentation is achieved well, it cannot remove all fragments. Delakas et al. reported that additional endourological procedures are carried out in 17% of patients who underwent SWL for bladder stone [9].

According to a study that compared the two aforementioned treatments, open surgery can completely remove stones, but it requires longer hospitalisation compared to SWL [10]. In contrast to open surgery, SWL only requires short hospitalisation time. However, patients treated with SWL may have residual fragments.

Endourological treatment is safe and effective alternative treatment for bladder stone. This treatment was first reported in 1963 [11]. Transurethral cystolithotripsy, percutaneous cystolithotripsy [12], and combined surgery with the two [13] have been reported with regard to how to access bladder stones. The percutaneous procedure is quite effective even for large bladder stones [13]. However, this procedure must be considered carefully for patients with history of bladder cancer, prior pelvic irradiation, abdominal or pelvic surgery, pelvic prosthesis, or active abdominal infection [14]. We prefer transurethral access to percutaneous access as the former is speculated to be safer for patients of various backgrounds.

In endourological treatment, several modalities are available, including ultrasonic lithotripsy, electrohydraulic lithotripsy, pneumatic lithotripsy, and a holmium:yttrium aluminium garnet (Ho:YAG) laser, to integrate stones [15]. Maheshwari, who first described the present method, used ultrasonic lithotripsy [1]. Ho:YAG laser is more effective than other modalities in treating bladder stones [15, 16]. Therefore, we utilised Ho:YAG laser. The optimal laser setting is unknown. Kawahara et al. previously reported that the power setting of Ho:YAG laser is not different from the operation time for bladder stone lithotripsy [17]. The author stated that laser frequency seems to affect operation time but lowers visualisation. Using an AS presses this drawback. The 6 Fr difference in diameter between 30 Fr AS and 24 Fr cystoscope results in more supply of irrigation flow under low intravesicle pressure, thereby enabling clear visualisation during operations. Therefore, in the AS group, surgeons can perform operation with strong laser setting and higher frequency than the SP group, which greatly reduced operation time and ensured safety.

The limitations of this study are as follows. First, the number of patients enrolled in this study was 18; this sample size may not be adequate to reach a definite conclusion. However, the difference between the two groups was remarkable, indicating that the AS affects cystolithotripsy. Second, the recurrence rate was poorly investigated. Patient follow-up after discharge was poor because 13 of 18 patients had general diseases that affected performance status. Thus, most of these patients had difficulty in visiting the hospital. We need further investigation with regard to recurrence of bladder stone after using this method.

## 5. Conclusion

Using an AS can shorten the operation time of cystolithotripsy without increasing perioperative complication.

## Figures and Tables

**Table 1:** Patients' characteristics (*N* = 18).

	AS group	SP group	*P* value
*N*	10	8	
Age (years old)	77.33 (63–91)	75.59 (64–83)	1.00
Stone volume (mL)	25.92 (9.84–99.63)	31.05 (17.43–73.002)	0.637
Stone burden (mm)^*∗*^	43 (36–133)	61 (30–130)	0.637
Number of stones	1 (1–4)	1 (1–4)	0.691
Neurological disease	7	7	0.375
History of febrile UTI^*∗∗*^	3	2	0.814
Preoperative pyuria	10	8	—

^*∗*^Stone burden: sum of the stones' maximum diameter; ^*∗∗*^UTI: urinary tract infection.

**Table 2:** Comparison of outcomes between these two groups.

	Group AS	Group SP	*P* value
*N*	10	8	
Stone-free	10	8	—
Total energy (kJ)	62.93 (12.50–166.55)	27.64 (5.12–44.47)	0.015
Operation time (min.)	58.50 (40–80)	112 (44–193)	0.015
Admission after operation (days)	5 (2–17)	4.5 (3–47)	0.480

Stone analysis			

Struvite (%)	8 (80)	6 (75)	0.493
Calcium phosphate (%)	1 (10)	0 (0)	
Mixed (%)	1 (10)	2 (25)	

Complication			

Postoperative pain	0.5 (0–3)	1 (0–2)	0.793
Fever > 38.0°C (%)	0 (0)	1 (12.5)	0.250
Mucosal injury (%)	0 (0)	0 (0)	—
Incontinence	0 (0)	0 (0)	—
